# The Influence of the COVID-19 Pandemic on Hospitalizations for Ambulatory Care-Sensitive Conditions in Split-Dalmatia County, Croatia

**DOI:** 10.3390/medicina60040523

**Published:** 2024-03-22

**Authors:** Ivana Marasović Šušnjara, Marijana Mijaković, Anamarija Jurčev Savičević

**Affiliations:** 1 Teaching Public Health Institute of Split-Dalmatia County, 21000 Split, Croatia; ivana.marasovic.susnjara@nzjz-split.hr (I.M.Š.); marijana.mijakovic@nzjz-split.hr (M.M.); 2 University Department of Health Studies, University of Split, 21000 Split, Croatia; 3 School of Medicine, University of Split, 21000 Split, Croatia

**Keywords:** COVID-19, ambulatory care-sensitive conditions, Croatia

## Abstract

*Background and Objectives:* We aimed to explore whether the COVID-19 pandemic influenced hospitalizations for ambulatory care-sensitive conditions (ACSCs) in Split-Dalmatia County, Croatia. *Materials and Methods*: We performed a cross-sectional comparative study using two different time periods, the pre-pandemic (from March 2019 to February 2020) and the pandemic period (from March 2020 to February 2021), to explore the possible influences that the COVID-19 pandemic had on hospitalizations for ACSCs. The ACSCs were classified into the categories of vaccine-preventable, chronic, and acute disease. The indicators were statistically analyzed. *Results*: During the pandemic, a decrease in the total number of hospitalizations and ACSC hospitalizations was recorded. The relative risk for having any ACSC hospitalization in the pandemic period compared to the pre-pandemic period was 0.67 (95% CI, 0.64–0.71; *p* = 0). The risk reduction was seen in all three categories of vaccine-preventable ACSCs, chronic disease, and acute disease. Large reductions were found in the relative risk of hospitalization for COPD and asthma. Considering the mode of discharge, there was a statistically significantly higher risk of ACSCs with fatal outcomes during the pandemic than in the pre-pandemic period (relative risk 1.31; 95% CI, 1.01–1.7; *p* = 0.0197). *Conclusions*: The results of this study show that the COVID-19 pandemic influenced the total number of hospitalizations as well as hospitalizations relating to ACSCs. Certainly, one of the reasons for these changes was due to organizational changes in the working of the entire health system due to the COVID-19 pandemic.

## 1. Introduction

The newly emerged SARS-CoV-2 spread very quickly, leading to the COVID-19 pandemic, as declared by the World Health Organization on 11 March 2020 [1]. In Croatia, the first case of infection was confirmed on 25 February 2020, and during the first year of the pandemic, 237,725 detected cases (58,481 per 1,000,000 inhabitants) and 5339 deaths (1313 per million inhabitants) were confirmed [2].

The burden of COVID-19 placed extreme pressure on health systems [3]. In particular, the demand for tertiary care grew. In order to respond to the increased demand, many countries, including Croatia, implemented a series of anti-epidemic measures. Reducing planned admissions to hospitals, earlier discharges, limiting outpatient visits, and online consultations are just some of the measures taken in order to successfully manage the pandemic [2]. In parallel with the implementation of these measures, around the world, concern was growing within the scientific community and wider society about possible negative consequences and the impact of the novel coronavirus on the quality of care of non-COVID-19 patients at all levels of health care [4,5,6,7].

Thus, during the initial pandemic-related closures, there was a sharp decrease in visits and admissions for other health care cases unrelated to COVID-19, including emergencies such as myocardial infarction and stroke [8]. A decline in the use of preventive care services, such as breast and cervical cancer screenings, sexually transmitted infection screenings, vaccinations, diabetes testing, and elective surgical procedures, was also found [2,9,10]. Very quickly, it became clear that the COVID-19 pandemic had a strong impact on all areas of the health system, not only infectious diseases and intensive care.

One way to measure health care quality is to use quality indicators. A widely used indicator is hospital admissions for so-called ambulatory care-sensitive conditions (ACSCs) [11]. ACSCs are conditions for which the need for hospitalization may be avoided by adequate management, treatment, and interventions delivered in the outpatient setting [11]. The concept of analyzing potentially avoidable hospitalizations started in the United States in the 1990s to evaluate access to health services [12]. Since then, ACSCs have attracted a high level of interest from researchers and policymakers [12,13]. The WHO Regional Office for Europe has produced a working document summarizing the evidence for using ACSCs as an indicator of health care performance in the dimensions of access, quality, coordination, and efficiency [14]. Simplified, a lower number/rate of hospitalizations generally means that ambulatory care is working well. If the numbers are higher, it means that there could be a problem in terms of access, efficiency, or both [15]. The utility and information provided when using ACSCs as an indicator depend on the further characteristics of the context in which they are measured and influencing factors such as geographical characteristics, sociodemographic characteristics, and the model of care [16]. Potentially avoidable hospitalizations can be classified as chronic conditions, where effective care can prevent flare-ups; acute conditions, where early intervention can prevent more serious progression; and preventable conditions, where immunization and other interventions can prevent illness [17]. Regarding this last category, only vaccine-preventable conditions are analyzed in ACSC research. Namely, there are subcategories of avoidable hospitalizations, comprising the hospitalization of people due to diseases that are preventable through population-based health-promotion strategies (e.g., alcohol-related conditions and most cases of lung cancer) and potentially avoidable hospitalizations through injury prevention strategies (e.g., road traffic accidents). However, currently, no approach has been agreed upon concerning the categorization of these aspects of avoidable hospitalizations across the world [18]. ACSC hospitalization rates are a well-established parameter for assessing the performance of primary health care (PHC). Although this indicator has been extensively used to monitor the performance of PHC systems under normal conditions, its consideration during disasters has been neglected [19]. In a recent study, the authors proposed that ACSCs can serve as a useful indicator of PHC performance during disasters, with several caveats that must be considered [19]. This suggestion is supported by some studies conducted in recent years that investigated the impact of the COVID-19 pandemic on ACSCs. Leuchter et al., (2021) compared a 6-month period in 2019 with the same 6-month period in 2020 and found a decrease in hospitalization rates for ACSCs during the COVID-19 pandemic [20]. Becker et al., (2022) compared hospitalization rates before the COVID-19 pandemic (March 2019–February 2020) and during the pandemic period (March 2020–February 2021), finding an overall reduction in all hospitalizations (both non-ACSCs and ACSCs), with a greater reduction for respiratory-related ACSC hospitalizations [9]. Wright et al. (2021) explored health care use and costs among different types of Medicaid enrollees before and during the COVID-19 pandemic, finding that new Medicaid enrollees during the COVID-19 pandemic were less likely to be hospitalized for ACSCs than those who enrolled before the COVID-19 pandemic [21]. Rennert-May et al. (2021) compared the most frequent ACSC diagnoses for hospital admissions before and after the implementation of COVID-19 public health measures to determine the impact of COVID-19 on hospital admissions and found that, although hospital admissions did not vary significantly, there was a significant reduction in admissions for chronic respiratory conditions [22].

The goal of this research was to provide more objective insight into the functioning of health care at the primary level with the requirements of a direct response to COVID-19. To our knowledge, no previous studies have investigated this topic in Croatia, and we have been the first to address the following research questions: (i) how the total number of admissions for ACSCs changed during the first year of the pandemic and (ii) which conditions most often lead to unplanned hospital admissions that were potentially preventable by proper management in the scope of primary care.

The results of this study will be useful for better understanding of how the COVID-19 pandemic has affected the health care system in terms of ACSCs. They can be used as an indicator to assess the adequacy, efficiency, and quality of primary health care within the broader health care system. They provide insight into the leading ACSCs and enable work on reducing their frequency and improving the related services. Ultimately, this may contribute to improving the health and quality of life of individuals and their families, reducing the burden on the health care system, improving the planning of health services, and reducing the negative impact on health financing at all levels. In addition, the results of this study may be useful in the management of health care and the implementation of measures in existing as well as in future epidemics and other crisis threats.

## 2. Materials and Methods

In this retrospective cross-sectional study, according to the Strengthening the Reporting of Observational Studies in Epidemiology (STROBE) reporting guidelines [23], we analyzed anonymized hospitalization data from the University Hospital of Split divided into two consecutive twelve-month periods. The first period, the pre-pandemic period, was from 1 March 2019 to 29 February 2020, and the second, the pandemic period, was from 1 March 2020 to 28 February 2021. We used data of admission as the criterion for inclusion. Out of all hospitalizations in these periods, we included only the inpatient hospitalizations of Split-Dalmatia County residents, which we identified from the ‘County of residence’ field in the National Public Health Information System database. We excluded the data from the Daily Hospital. The data on the main discharge diagnosis from the ICD-10 Chapter XV Pregnancy, childbirth, and the puerperium (O00–O99), as well as Outcome of the delivery (Z37) from Chapter XXI were also excluded from this analysis.

Hospitalizations were categorized as an ambulatory care-sensitive condition (ACSC) hospitalization, COVID-19 hospitalization, and the rest, namely non-ACSC and non-COVID hospitalizations. ACSC hospitalizations were identified using the list based on the Australian work by Vic DHS and subsequent development by NSW Health [18]. In this research, we used this list because Croatia has not yet established its own ACSC definition and because some previous studies in Croatia have used it [15,24]. According to the mentioned list, ACSC hospitalizations were divided into three categories: vaccine-preventable ACSC hospitalizations, chronic ACSC hospitalizations, and acute ACSC hospitalizations. The vaccine-preventable category was further divided into the influenza and pneumonia group, and the other vaccine-preventable hospitalizations group (other tetanus, diphtheria, whooping cough, acute poliomyelitis, measles, rubella, acute hepatitis B with delta-agent (coinfection) without hepatic coma, acute hepatitis B without delta-agent and without hepatic coma, chronic viral hepatitis B with delta-agent, chronic viral hepatitis B without delta-agent, mumps, haemophilus meningitis, rubella arthritis). The chronic category was further divided into the following groups: diabetes complications, nutritional deficiencies, iron deficiency anemia, hypertension, congestive heart failure, angina, chronic obstructive pulmonary disease, and asthma. The acute category was further divided into: de-hydration and gastroenteritis, convulsions and epilepsy, ear, nose, and throat infections, dental conditions, perforated/bleeding ulcer, ruptured appendix, pyelonephritis, pelvic inflammatory disease, cellulitis, and gangrene. The categories, associated diseases, and their corresponding ICD-10 codes are provided in Table 1.

We used the estimation of the number of Split-Dalmatia County residents on 30 June from the Croatian Bureau of Statistics PC-Axis Databases Population estimate, divided by age groups and sex as well as by county, to calculate the relative risk for Split-Dalmatia County residents of having any hospitalization, ACSC hospitalization, non-ACSC non-COVID hospitalization, or hospitalization from each of the defined categories and groups within the pre-pandemic period compared to the pandemic period. For this calculation, we assumed that each hospitalization reduces the number of non-hospitalized Split-Dalmatia County residents by one.

We calculated the relative risk for a hospitalization to be an ACSC hospitalization out of all other possible hospitalizations, the relative risk of each of the categories and groups of the ACSC hospitalizations, and the relative risk for age group-specific ACSC hospitalizations in the pre-pandemic period compared with the pandemic period.

To check if the type of discharge of the ACSC hospitalizations in the pre-pandemic and pandemic periods differs significantly, we divided all hospitalizations into two groups based on the type of discharge, namely ‘Death’ and ‘All the other types of the discharge’. We used the χ^2^ test of independence [25] and we also calculated the relative risk of death as the discharge method for the ACSC hospitalizations in the pandemic period relative to the pre-pandemic period. All the relative risks were calculated with the relative risk calculator [26].

To check if there was a difference in hospitalizations between the pre-pandemic and pandemic periods, we divided hospitalizations into three age groups, namely 0–19 years, 20–64 years, and 65 or more years. The χ^2^ test of independence was used to check if the distribution of hospitalizations by the age group of the hospitalized person is independent of the season, and to check if the distribution of the monthly number of all hospitalizations and ACSC hospitalizations is independent of the season [25].

Two-tailed *t*-test with assumption of unequal variances was used to check the mean difference between the number of all monthly and ACSC monthly hospitalizations in two periods. To test the normality of the inpatient length of stay data in both periods, we performed the Kolmogorov–Smirnov test of normality [27]. To compare the means of the inpatient lengths of stay of ACSC hospitalizations in these two periods we used a Two-tailed Mann–Whitney U test on random samples from both periods [28]. The size of the sample for the pre-pandemic period was 340 and for the pandemic period it was 320. A *p*-value of less than 0.05 was accepted as indicating statistical significance.

## 3. Results

### 3.1. All Hospitalization’s General Descriptive Data and Age Distribution

In the pre-pandemic period (from March 2019 to February 2020), we registered 35,889 hospitalizations of Split-Dalmatia County residents at the University Hospital of Split. A total of 2722 (7.6%) of them were ACSC hospitalizations. In this period there were no COVID-19-related hospitalizations. In the pandemic period (from March 2020 to February 2021), out of 28,137 hospitalizations of Split-Dalmatia County residents at the University Hospital of Split, 1827 (6.5%) were ACSC hospitalizations, and 1938 (6.9%) were COVID-19-related hospitalizations. In the pre-pandemic period, 51.5% of all of the hospitalizations were hospitalizations of males, and 48.5% of females. In the pandemic period, 52.8% of all hospitalizations were of males, and 47.2% of females. Table 2 reports the distribution of all hospitalizations by the age of the hospitalized person in the pre-pandemic and pandemic periods.

We found that the distribution of hospitalizations by the age of the hospitalized person differs in the pre-pandemic and pandemic periods (χ^2^ = 187.8, *p* = 0). In the pandemic period, there were less hospitalizations of persons from younger age groups than in the pre-pandemic period.

### 3.2. Timeline of All Hospitalizations, ACSC Hospitalizations, and COVID-19 Hospitalizations

**Figure 1:**
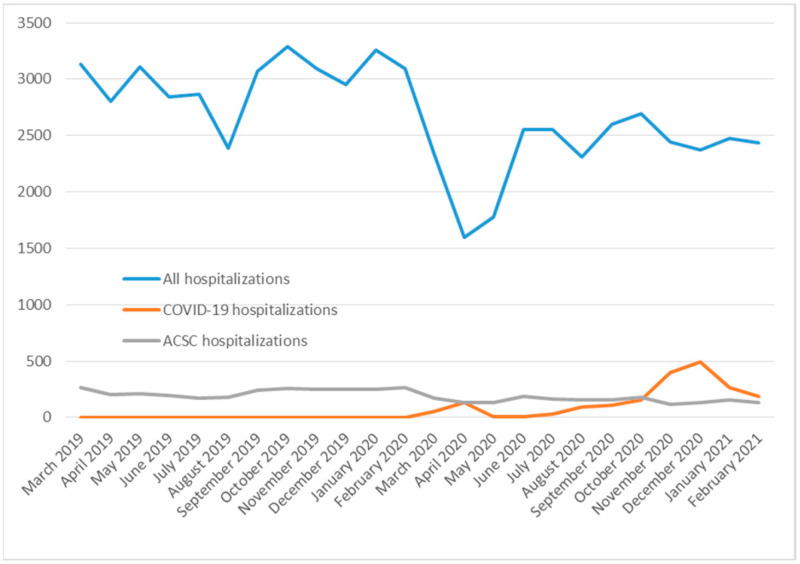
Timeline of all, COVID-19, and ACSC hospitalizations in the pre-pandemic and pandemic periods.

In Figure 1, we show the number of all hospitalizations, COVID-19 hospitalizations, and ACSC hospitalizations distributed by the month of the date of admission. There is a visible drop in the number of all hospitalizations and ACSC hospitalizations in the pandemic period.

The number of monthly hospitalizations has a different distribution in these two periods (χ^2^ = 337.1, *p* = 0.000001). There is an obvious drop in the monthly number of all hospitalizations in April and May in the pandemic period. In the pre-pandemic period, the number of hospitalizations in the October–February period is higher than in the June–August period. However, in the pandemic period, we see almost no difference in the monthly number of hospitalizations in the June–August period compared to the October–February period. The mean value of the monthly number of hospitalizations in the pre-pandemic period is 2990, while it is 2345 in the pandemic period (*t*-test = 5.4, *p* = 2.5 × 10^−5^).

For the Split-Dalmatia residents, the relative risk of having any hospitalization in the pandemic period compared to the pre-pandemic period was found to be 0.79 (95% CI, 0.78–0.80, *p* < 0.001) (Table 3).

### 3.3. ACSC Hospitalizations

The distribution of ACSC hospitalizations by the age group of the hospitalized person was found to be independent of the season (pre-pandemic or pandemic period) (χ^2^ = 5.33, *p* = 0.07). Looking at the relative likelihood of the hospitalization to be an ACSC hospitalization as opposed to all other types of hospitalization, we found no difference in the 0–19 age group in these two periods. However, in the 20–64 age group, we found that the relative likelihood of a hospitalization to be an ACSC hospitalization in the pandemic period compared to the pre-pandemic period was 0.82 (95% CI, 0.74–0.91, *p* = 0.0001). Additionally, in the 65 or more age group, we found the relative likelihood of a hospitalization to be an ACSC hospitalization in the pandemic period compared to the pre-pandemic period to be 0.88 (95% CI, 0.77–0.92, *p* < 0.0001).

**Figure 2:**
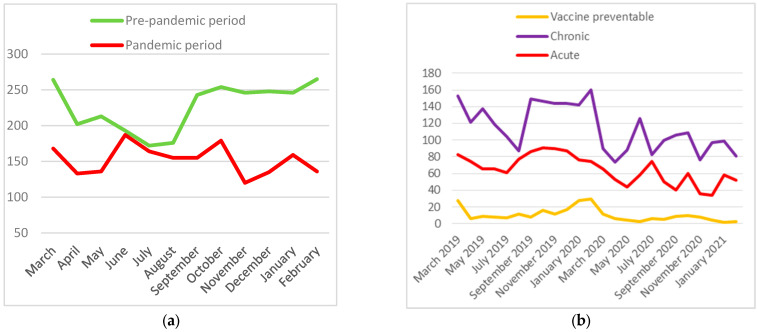
(**a**) Number of ACSC hospitalizations, (**b**) timeline of three categories of ACSC hospitalizations in pre-pandemic and pandemic periods.

The mean value of the monthly number of ACSC hospitalizations in the pre-pandemic period is 227, while it is 152 in the pandemic period (*t*-test = 6.5, *p* = 3.8 × 10^−6^). The number of monthly ACSC hospitalizations has different distribution in these two periods (χ^2^ = 49.3, *p* = 0.000001). In the pre-pandemic period, we see a drop in the monthly number of ACSC hospitalizations from April to August, while in the pandemic period the number of ACSC hospitalizations exhibits no such a drop. On the contrary, it shows a slight rise in the period from June to October (Figure 2a).

The relative risk of having an ACSC hospitalization in the pandemic period compared to the pre-pandemic period was found to be 0.67 (95% CI, 0.64–0.71, *p* < 0.001) (Table 3).

The relative risk of having a non-ACSC, non-COVID-19 hospitalization in the pandemic period compared to the pre-pandemic period was found to be 0.74 (95% CI, 0.73–0.75, *p* < 0.001) (Table 3).

### 3.4. Vaccine-Preventable, Chronic and Acute ACSC Hospitalizations Distribution and Relative Risks

In Figure 2b, we show the number of vaccine-preventable, chronic, and acute ACSC hospitalizations. In the pre-pandemic period, vaccine-preventable ACSC hospitalizations accounted for 6.6% of all ACSC hospitalizations, chronic ACSCs for 59.1%, and acute ACSCs for 34.3%. However, this distribution of the ACSC categories significantly changes in the pandemic period (χ^2^ = 15.8, *p* = 0.0004). The percentage of vaccine-preventable ACSC hospitalizations drops and of the number chronic ACSC hospitalizations rises. The percentage of acute ACSC hospitalizations was found to be approximately the same in both periods. In the pandemic period, vaccine-preventable ACSC hospitalizations accounted for 3.9%, chronic ACSCs for 61.8%, and acute ACSCs for 34.3% of all ACSC hospitalizations. Chronic ACSC hospitalizations were in the majority in both observed periods.

For the Split-Dalmatia County residents, the relative risk of having a vaccine-preventable ACSC hospitalization in the pandemic period compared to the pre-pandemic period was found to be 0.40 (95% CI, 0.30–0.52, *p* < 0.001). The relative risk for having a chronic ACSC in the pandemic period compared to the pre-pandemic period was found to be 0.70 (95% CI, 0.65–0.76, *p* < 0.001). The relative risk for having an acute ACSC in the pandemic period compared to the pre-pandemic period was found to be 0.67 (95% CI, 0.61–0.75, *p* < 0.001).

### 3.5. Vaccine-Preventable, Chronic and Acute ACSC Subcategories Hospitalizations

When we look at the vaccine-preventable ACSC hospitalizations of Split-Dalmatia County residents, we found that, for the influenza and pneumonia category, the relative risk for having a hospitalization in the pandemic period compared to the pre-pandemic period was 0.42 (95% CI, 0.32–0.55, *p* < 0.001). For the other vaccine-preventable hospitalizations, it was found to be 0.09 (95% CI, 0.01–0.71, *p* = 0.01).

In Table 4, we show significant relative risks for the chronic, acute, and vaccine preventable ACSC categories in the pandemic period compared to the pre-pandemic period.

For the nutritional deficiencies category, iron deficiency anemia category, and congestive heart failure category, there was no significant difference in these two periods. For the pelvic inflammatory disease category, the risk of hospitalization was bigger in the pandemic period than in the pre-pandemic period. For the ear, nose, and throat infection category, dental conditions category, ruptured appendix category, and gangrene category, there was no significant difference in the pandemic and pre-pandemic periods for Split-Dalmatia County residents.

As previously stated, in both observed periods, chronic ACSC hospitalizations were in the majority. Specifically, the chronic ACSC sub-category congestive heart failure made up 17.4% of all ACSC hospitalizations in the pre-pandemic period, and 23% in the pandemic period. Diabetes complications made up 14.6% and angina 14.5% in the pre-pandemic period. In the pandemic period, angina made up 15.2% and diabetes complications 12.6% of the ACSC hospitalizations.

### 3.6. Type of Discharge Analyses

Further, we analyzed the ‘Type of discharge’ for the ACSC hospitalizations (Table 3). We found that there was significantly greater relative risk for ACSC hospitalization to end in ‘Death’ as the type of discharge in the pandemic period compared to the pre-pandemic period, RR = 1.31 (95% CI, 1.01–1.70, *p* = 0.02).

Next, we analyzed whether the type of discharge depends on the ACSC category of hospital admission. We found that, in the pre-pandemic period, the type of discharge is dependent on the ACSC category (χ^2^ = 24.9, *p* = 0.000004). In the pre-pandemic period, 5.7% of chronic ACSC hospitalizations ended in death. In the same period, 5.5% of vaccine-preventable and 1.6% of acute ACSC hospitalizations ended in death. In the pandemic period, we also found that the type of discharge is dependent on the ACSC category (χ^2^ = 35.5, *p* < 0.000001). However, in the pandemic period, vaccine-preventable ACSC hospitalizations had the largest percent of death as the type of discharge, namely 11.1% of vaccine-preventable ACSC hospitalizations ended in death. For the same period, 7.7% of chronic and 1.3% of acute ACSC hospitalizations ended in the death type of discharge.

### 3.7. Inpatient Length of Stay Analyses

Inpatient length of stay of ACSC hospitalizations data follow the general shape of the length of stay data, meaning that they have a highly right-skewed distribution. The length of stay of ACSC hospitalization data distributions were first tested for normality. The results, which we show in the Supplementary Materials, provide evidence that length of stay data were not normally distributed (Figures S1–S4).

Our results indicate a significant difference between the inpatient lengths of stay for ACSC hospitalizations (U = 48,295.5, *p* = 0.01) in the pre-pandemic and the pandemic period, and for acute ACSC hospitalizations (U = 28,667, *p* = 0.02). The mean length of stay for ACSC hospitalizations in the pre-pandemic period was 7.6 days, while it was 6.9 days in the pandemic period. For acute ACSC hospitalization, the mean length of stay in the pre-pandemic period was 7.3 days, and 6.8 in the pandemic period.

For the vaccine-preventable ACSC hospitalizations (U = 6096, *p* = 0.424) and chronic ACSC hospitalizations (U = 42,402.5, *p* = 0.294), we found no significant difference between the inpatient lengths of stay in the pre-pandemic and the pandemic period. The mean length of stay for vaccine-preventable ACSC hospitalizations in the pre-pandemic period was 8.5 days, while it was 8.7 days in the pandemic period. For the chronic ACSC hospitalizations, the mean length of stay in the pre-pandemic period was 7.2 days, and 7.2 in the pandemic period.

## 4. Discussion

### 4.1. Principal Findings

The research results showed a reduction in non-ACSC and ACSC hospitalizations in total in the pandemic period compared to the pre-pandemic period. There was a reduction in all ACSC categories, acute, chronic, and vaccine-preventable, although the reduction in the category of chronic diseases contributed the most, due to the reductions in vaccination-preventable-related respiratory disease hospitalizations. Additionally, the study found that, in both of the observed periods, chronic ACSCs made up the majority of the ACSC hospitalizations. In both periods, congestive heart failure was found to be the most common cause of ACSC hospitalization, followed by diabetes complications and angina. Additionally, this research did not establish that the duration of hospitalization was extended during the COVID-19 pandemic, but a higher risk of fatal ACSC hospitalization outcomes during the pandemic period was determined.

### 4.2. Comparisons with Other Studies

The results of our research are in line with several previously conducted studies that considered the impact of the COVID-19 pandemic on ACSC hospitalizations [9,22,29].

As there was a decrease in both non-ACSC and ACSC hospitalizations during the observed period, Becker and the authors believe that there should be caution in interpreting the results, which would indicate the decrease in ACSC hospitalizations solely as a result of the quality of ambulatory care. Rather, it seems likely that the pandemic created many changes in patient behavior patterns and access to care that may have been associated with ACSC hospitalization rates, even in the absence of a change in the quality of ambulatory care delivery [9].

The reduction in the number of hospitalizations that are not related to COVID-19 in some countries was argued to be caused by the increased influx of patients with COVID-19, and the implemented anti-pandemic measures within health systems. In this study, we believe that the significant reduction in the number of hospitalizations at the beginning of the pandemic is a consequence of the introduced national anti-pandemic measures [30], which is supported by some other Croatian research [31,32]. Influenced by the negative experience of countries that suffered the worst consequences at the beginning of the pandemic, many countries, including Croatia, introduced rigorous preventive measures, the so-called lockdown [33]. To prevent the spread of the virus, the initial response was to close the borders and suspend international flights. Depending on the development of the pandemic, more restrictive measures of isolation/self-isolation, quarantines, temporary closure of educational institutions, restrictions on social gatherings, restrictions of travel and movement at the local level, and self-protection measures were implemented [34]. In this sense, the health care system has undergone a series of adjustments. In certain hospitals, wards or outpatient facilities were reassigned as COVID-19 facilities, while other hospitals established COVID-19 isolation wards. With this reorganization, 15% of hospital beds in Croatia were designated as COVID-19 beds [34]. The work of the hospital staff was also reorganized, into shifts of 2 weeks. The introduction of shift work in hospitals also resulted in a reduction in hospital outpatient consulting hours, which most likely had an impact on the admission rates, given that non-emergency admissions in Croatia are initiated by hospital-based specialists in an outpatient setting [31]. According to the revised admission procedures, only the patients who tested negative for COVID-19 were admitted to the general wards. The reorganization of the hospital system reduced access to health services at all levels of health care (primary, secondary, and tertiary), except for emergency patients, oncology patients, pregnant women, and those suffering from COVID-19. In addition to regular health services, certain preventive programs were also suspended. Overall, regular patient procedures were drastically reduced from March 2020 onwards [32]. To ensure access to health services for all patients who were not affected by COVID-19, new digital health solutions and services were established, targeting the general population and, specifically, vulnerable groups. These new services included websites, mobile apps, and phone lines. In addition, some health institutions or non-governmental organizations established telephone lines or internet consultations for patients and organized periodic field visits to members of vulnerable groups [32]. The decrease in the chronic diseases category, that is, the decrease in the number of hospitalizations for vaccine-preventable respiratory diseases, can be explained by multiple reasons, including the behavior of the patients themselves regarding their disease management. The authors of the conducted studies attribute the decrease in hospitalizations of patients with asthma during the COVID-19 pandemic to a positive increase in adherence to prescribed therapy [35]. On the other hand, the doctors’ attitudes towards these patients, who are high-risk groups where care was especially emphasized during the pandemic, could also have contributed to the reduction. We cannot rule out the possibility that the decrease in the number of hospitalizations in this group could also be connected to their potential categorization as having COVID-19. However, a number of other factors could also contribute to the reduction as a result of rigorous measures introduced during the pandemic, such as traveling restrictions resulting in air pollution reduction or the reduced circulation of respiratory viruses [35]. We also recorded a somewhat smaller decrease in other categories of chronic ACSC hospitalization, such as diabetes. We agree with the authors that healthy lifestyle habits, maintenance, or dietary habit improvements in people with diabetes, even during the period of stay-at-home, could have contributed to preventing an increase in the number of admissions in this ACSC category, and could be partly responsible for the reduction in other ACSC hospitalization categories as well [29,36].

Furthermore, changes in individual lifestyle conditioned by mandatory measures [34] such as the mandatory wearing of masks or more frequent hand washing could contribute to the reduction in acute ACSCs, which was recorded for almost all conditions, except for pelvic inflammatory diseases. The reason for the increase in pelvic inflammatory disease, as already mentioned, could be the delayed presentation and progression to more severe disease in some patients. This is also the opinion of some other authors [33]. Generally, the SARS-CoV-2 (COVID-19) pandemic is associated with numerous disruptions in routine medical care.

Through research, we have also shown a reduction in the category of preventable vaccination-avoidable hospitalizations, which are mainly related to influenza and pneumonia, which are among the main ACSCs that lead to hospital admissions [37]. Managing these conditions is extremely important, especially in situations where the demand for hospital services increases, as was the case due to the COVID-19 pandemic because hospitalizations related to SARS-CoV-2 put pressure on the health care system. Overall, effective interventions for conditions such as influenza and pneumonia are to support primary care management, which can help to reduce admissions for diseases which can be prevented. According to some analyses, vaccine-related interventions can lead to a significant reduction in hospital admissions as well as the number of hospital days [37].

During the pandemic period, a higher risk of death outcomes was found in ACSC hospitalizations. This is especially evident in vaccine-preventable ACSC hospitalizations. Increased in-hospital mortality rates of ACSC-related hospitalizations during the COVID-19 pandemic have also been reported by other authors [38]. On the one hand, this could suggest that patients, for some reason, did not seek help on time, as was already shown for acute coronary syndrome [39,40]. It is possible that patients delayed seeking help because they were afraid that they might get infected when visiting health institutions, as confirmed by patients themselves in previously published reports [4,40]. In addition, a large number of services in primary care were provided remotely, which could affect the care availability for some categories of patients. However, it is also possible that the severity of their illness was such that it would have resulted in death despite everything. As it is already known, poor health is a risk factor for hospitalization [41].

It was not found that the duration of hospitalization was prolonged during the COVID-19 pandemic, which suggests that people did not need more complex medical care after being hospitalized. On the contrary, we found that, for ACSC hospitalizations, and especially for acute ACSC hospitalizations, the length of stay was shorter in the pandemic period, which may suggest more efficient medical care. With the combination of other factors, such as attempts to diminish possible patient exposure to the SARS-CoV-2 virus in the hospital environment, we may explain the shortening of the inpatient length of stay in the pandemic period.

Therefore, although our results suggest that there were probably no major reductions in the quality of ambulatory care during the pandemic, we must keep in mind and take into account the limitations related to the use and complexity of interpreting the ACSC indicators due to more comprehensive effects on health needs, which other authors also consider [9,29]. The purpose of the concept of ACSCs is to indicate the morbidity target categories that reflect the weak points of the population’s health and to evaluate the effects of individual activities. The possibility of designing and implementing health interventions that can lead to an improvement in the health care system should be highlighted. Countries that have applied this concept have confirmed that the early identification, intervention, and management of disease plays a major role in preventing the appearance or worsening of the disease and/or reducing the hospitalization rate [42]. Discussions about the possibilities of reducing ACSCs are aimed toward the new paradigm concerning health care priorities and resulting in efforts to invest in specific preventive programs for specific health problems or for specific population groups.

### 4.3. Strengths and Limitations

The importance of our research is that it contributes to the knowledge of which conditions most often lead to potentially preventable unplanned hospital admissions, and which can be managed at the primary care level. This could potentially free up space in hospitals for the needs of patients as a result of the pandemic and, on the other hand, help protect patients with acute and chronic diseases who would not be unnecessarily exposed to infection in hospitals, while their condition would be effectively treated at the level of primary health care. In the context of limited resources and increasing health expenditure, the possibility of decreasing spending by avoiding unnecessary or inappropriate hospitalizations is relevant to national health agendas worldwide [43].

It is necessary to point out some limitations of this research. Given that this study uses routinely collected administrative data that were not primarily created for the purposes of this research, their potential shortcomings should be taken into account when interpreting the results. Additionally, the indicator used has not been confirmed for Croatia, although it has been used by different authors as an indirect measure for assessing different aspects of the health care system, whether in studying access to or the quality of health care [24]. Furthermore, the use of this indicator as an indicator of quality in primary health care requires caution, because the results can be influenced not only by the ability to reduce health problems in primary health care, but also by other variables, such as morbidity, different patterns of use of health resources, and the use of specialist care [44]. In addition, this study was conducted in one center, so the results may not be generalizable to the health care system as a whole.

## 5. Conclusions

The results of our study, as well as of several others around the world, showed that during COVID-19 there was a decrease in the number of hospitalizations in all ACSC categories: chronic, acute, and vaccine-preventable. However, as there was a decrease in the total number of hospitalizations, we cannot attribute the observed decrease only to the level of ambulatory care, and must also include other factors at the levels of health policy, the health system, and individuals.

Additionally, this study found that, in both of the observed periods, chronic ACSCs made up the majority of the ACSC hospitalizations. In both periods, congestive heart failure was found to be the most common cause of ACSC hospitalization, followed by diabetes complications and angina. Reducing hospitalizations for ambulatory care conditions presents a challenge for the health care system on all levels, including the policymakers level. This study implies the need for further action to increase the availability of primary health care and improveme continuous and coordinated care across the health care system.

## Figures and Tables

**Table 1:** Ambulatory care-sensitive conditions (ACSCs) and ICD-10 codes [18].

Category	ICD-10 Code
Vaccine-preventable	
Influenza and pneumonia	J10, J11, J13, J14, J153, J154, J157, J159, J168, J181, J188
Other vaccine preventable	A35, A36, A37, A80, B05, B06,B161, B169, B180, B181,B26,G000, M014
Chronic	
Diabetes complications	E101–E108, E110–E118, E130–E138, E140–E148
Nutritional deficiencies	E40–E43, E55.0, E643
Iron deficiency anemia	D501-D509
Hypertension	I10, I119
Congestive heart failure	I110, I50, J81
Angina	I20, I240, I248, I249
Chronic obstructive pulmonary disease	J41–J44, J47, (J20)
Asthma	J45, J46
Acute	
Dehydration and gastroenteritis	E86, K522, K528, K529
Convulsions and epilepsy	G40, G41, O15, R56
Ear, nose and throat infections	H66, H67, J02, J03, J06, J312
Dental conditions	A690, K02–K06, K08, K098, K099, K12, K13
Perforated/bleeding ulcer	K250–K252, K254–K256, K260–K262, K264–K266, K270–K272, K274–K276, K280–K282, K284–K286
Ruptured appendix	K35
Pyelonephritis	N10, N11, N12, N136
Pelvic inflammatory disease	N70, N73, N74
Cellulitis	L03, L04, L08.0, L08.8, L08.9, L88, L98.0, L98.3
Gangrene	R02

**Table 2:** Distribution of all hospitalizations by age of the hospitalized person in the pre-pandemic and pandemic periods.

Age Group	Pre-Pandemic Period	Pandemic Period
1–6	1072 (3.0%)	718 (2.6%)
7–14	1172 (3.3%)	794 (2.8%)
15–19	848 (2.4%)	577 (2.1%)
20–34	2825 (7.9%)	2132 (7.6%)
35–44	2482 (6.9%)	1888 (6.7%)
45–54	3620 (10.1%)	2739 (9.7%)
55–64	6081 (16.9%)	4795 (17.0%)
65–74	8054 (22.4%)	6731 (23.9%)
75 and more	8571 (23.9%)	7245 (25.7%)

**Table 3:** Summary statistics for relative risks.

	Pre-Pandemic Period (March 20– February 2020)	Pandemic Period (March 2020– February 2021)	Pandemic vs. Pre-Pandemic Relative Risk (95% CI)	*p*-Value
Hospitalization				
Total	35,889	28,137	0.79 (0.775–0.798)	<0.01
ACSC	2772	1872	0.67 (0.635–0.714)	<0.01
ACSC category				
Vaccine-preventable	181	72	0.51 (0.386–0.666)	<0.01
Chronic	1608	1129	0.90 (0.831–0.965)	<0.01
Acute	933	626	0.86 (0.774–0.946)	<0.01
ACSC—Type of discharge				
Death	117	103	1.31 (1.013–1.698)	0.05
Other (home or transfer to another institution)	2605	1724	0.986 (0.973–1.000)	0.02

ACSC: ambulatory care-sensitive conditions.

**Table 4:** Relative risks for hospitalization for the each of the chronic, acute, and vaccine-preventable ACSC categories in the pandemic period compared to the pre-pandemic period for the Split-Dalmatia County residents.

**ACSC Category**	**Relative Risk (95% CI)**	***p*-Value**
Chronic		
Diabetes complications	0.58 (0.49–0.68)	<0.001
Hypertension	0.63 (0.46–0.86)	0.004
Angina	0.71 (0.61–0.82)	<0.001
COPD	0.37 (0.26–0.51)	<0.001
Asthma	0.22 (0.09–0.54)	<0.001
Acute		
Dehydration and gastroenteritis	0.56 (0.36–0.88)	0.006
Convulsions and epilepsy	0.76 (0.63–0.91)	0.002
Perforated/bleeding ulcer	0.57 (0.44–0.74)	<0.001
Pyelonephritis	0.52 (0.40–0.67)	<0.001
Pelvic inflammatory disease	10.03 (2.35–42.92)	<0.001
Cellulitis	0.52 (0.38–0.72)	<0.001
Vaccine-preventable		
Influenza and pneumonia	0.42 (0.32–0.55)	<0.001
Other vaccine preventable	0.09 (0.01–0.71)	<0.001

ACSC: ambulatory care-sensitive conditions; COPD: chronic obstructive pulmonary disease.

## Data Availability

All the data used in our study are included in the article/Supplementary Materials, and further inquiries can be directed to the corresponding author.

## Supplementary Materials

The following supporting information can be downloaded at: https://www.mdpi.com/article/10.3390/medicina60040523/s1, Figure S1: Length of stay of ACSC hospitalization data distributions for the pre-pandemic period and the pandemic period; Figure S2: Length of stay of vaccine-preventable ACSC hospitalization data distributions for the pre-pandemic period and the pandemic period; Figure S3: Length of stay of chronic ACSC hospitalization data distributions for the pre-pandemic period and the pandemic period; Figure S4: Length of stay of acute ACSC hospitalization data distributions for the pre-pandemic period and the pandemic period.
