# Supplementary material for: The Influence of the COVID-19 Pandemic on Hospitalizations for Ambulatory Care-Sensitive Conditions in Split-Dalmatia County, Croatia
Source: Medicina (Kaunas). 2024 Mar 22;60(4):523. doi: 10.3390/medicina60040523 (PMC11052272; doi:10.3390/medicina60040523)
Supplement: Supplementary file 1 [file medicina-60-00523-s001.zip › Figure S1.pdf]

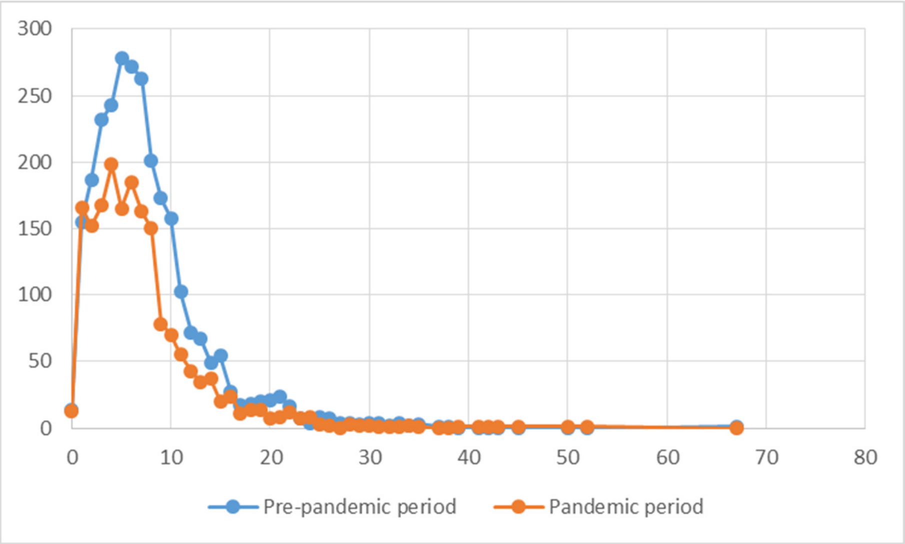

Figure S1. Length of stay of ACSC hospitalisation data distributions for the pre-pandemic period and the pandemic period.

A Kolmogorov-Smirnov test was used to test the normality of the Length of stay of ACSC hospitalisations data distribution. The results in both periods ($D = 0.17$, $p < 0.00001$ for the pre-pandemic period and $D = 0.15$, $p < 0.00001$ for the pandemic period) suggest that data are not normally distributed.
