# Supplementary material for: The Influence of the COVID-19 Pandemic on Hospitalizations for Ambulatory Care-Sensitive Conditions in Split-Dalmatia County, Croatia
Source: Medicina (Kaunas). 2024 Mar 22;60(4):523. doi: 10.3390/medicina60040523 (PMC11052272; doi:10.3390/medicina60040523)
Supplement: Supplementary file 1 [file medicina-60-00523-s001.zip › Figure S2.pdf]

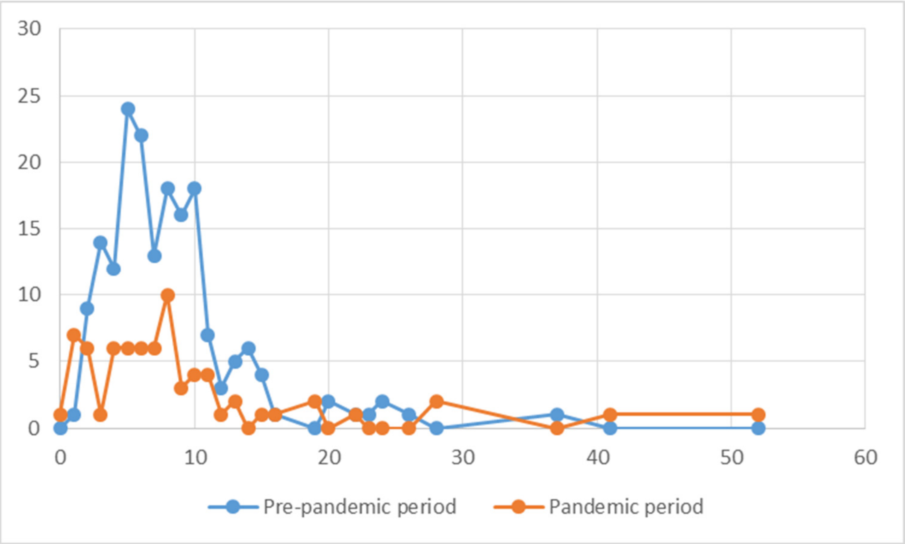

Figure S2. Length of stay of vaccine-preventable ACSC hospitalisation data distributions for the pre-pandemic period and the pandemic period.

A Kolmogorov-Smirnov test was used to test the normality of the Length of stay of vaccine-preventable ACSC hospitalisations data distribution. The results in both periods (D = 0.16, p = 0.00022 for the pre-pandemic period and D = 0.23, p = 0.00106 for the pandemic period) suggest that data are not normally distributed.
